# Inhibition of exosomal miR‐24‐3p in diabetes restores angiogenesis and facilitates wound repair via targeting PIK3R3

**DOI:** 10.1111/jcmm.15958

**Published:** 2020-11-03

**Authors:** Yan Xu, Liu Ouyang, Lei He, Yanzhen Qu, Yu Han, Deyu Duan

**Affiliations:** ^1^ Department of Orthopaedics Union Hospital Tongji Medical College Huazhong University of Science and Technology Wuhan China; ^2^ Department of Orthopaedic Surgery Shanghai Key Laboratory of Orthopaedic Implants Shanghai Ninth People’s Hospital Shanghai Jiaotong University School of Medicine Shanghai China

**Keywords:** human umbilical vein endothelial cells, miRNA, PIK3R3, wound repair

## Abstract

Diabetic foot ulcer (DFU) is one of the common ailments of elderly people suffering from diabetes. Exosomes containing various active regulators have been found to play a significant role in apoptosis, cell proliferation and other biological processes. However, the effect and the underlying mechanism of action of diabetes patients derived from circulating exosomes (Dia‐Exos) on DFU remain unclear. Herein, we aim to explore the potential regulatory role of Dia‐Exos. First, we attempted to demonstrate the harmful effect of Dia‐Exos both in vivo and in vitro. miRNA‐24‐3p (miR‐24‐3p) was found enriched with Dia‐Exos. Hence, inhibition of this miRNA could partially reverse the negative effect of Dia‐Exos, thus, in ture, accelerates wound repair. Luciferase assay further verified the binding of miR‐24‐3p to the 3′‐UTR of phosphatidylinositol 3‐kinase regulatory subunit gamma (*PIK3R3*) mRNA and the PIK3R3 expression enhanced human umbilical vein endothelial cells functionality in vitro. Hence, the findings of this study reveal the regulatory role of Dia‐Exos in the process of wound healing and provide experimental evidence for the therapeutic effects of knocking down miR‐24‐3p in DFU treatment.

## 1 INTRODUCTION

The incidence of diabetic foot ulcer (DFU) is continuously increasing in elderly diabetic patients. The treatment of DFU in elderly people is often unsatisfactory due to their relatively weak immunity and the vascular lesions caused by diabetes.^1^, ^2^ Furthermore, commonly associated diseases with DFU such as infection, gangrene and other serious complications potentially lead to unavoidable amputations and other destructive consequences both the patient’s physical and mental health, thereby posing serious threats to people’s health.^3^, ^4^ At present, there are various clinical treatments for DFU, and many new drugs have been developed for diabetic wound repair.^5^, ^6^, ^7^, ^8^ However, these treatments cannot provide maximum clinical outcome for DFU, and the specific molecular mechanism of DFU development remains elusive. Exosome, an extracellular vesicle with a diameter of 30‐100 nm, has a strong biological function. The vesicle contains non‐coding RNA, cholesterol, protein and other bioactive substances and has been extensively proved for regulating diverse biological processes in vivo.^9^, ^10^ In this study, circulating exosomes from diabetic (Dia‐Exos) and non‐diabetic patients (Con‐Exos) were isolated, extracted and identified, and their respective biological functions and underlying molecular mechanisms were further studied.

Angiogenesis, the process of new blood vessel formation, plays a vital role in wound healing.^11^ Diabetes‐associated peripheral vascular lesions could result in insufficient blood supply to the wound area and eventually lead to delayed wound healing.^12^ The vascular endothelial cells belong to an important class of cells in the process of angiogenesis.^13^ Recent research has confirmed that circulating exosomes derived from diabetes patients have adverse effects on the process of angiogenesis. However, the damage caused by diabetic circulating exosomes to the wound angiogenesis was significantly reversed by knocking down the exosomal miR‐20b‐5p expression. Thus, it is suggested that selective knockdown of the miR‐20b‐5p level in the diabetic circulating exosomes can effectively promote diabetic wound healing.^14^, ^15^

In the current work, we aimed at investigating the impact of Dia‐Exos on the functionality of human umbilical vein endothelial cells (HUVECs), wound healing as well as the underlying mechanism of this regulatory process, thereby providing potential therapeutic strategies for the treatment of DFU.

## 2 MATERIALS AND METHODS

### 2.1 Transfection and cell culture

Human umbilical vein endothelial cells were procured from the Cell Bank at the Chinese Academy of Science, Shanghai, China. An RPMI 1640 medium obtained from ThermoFisher Scientific with 10% exosome‐depleted foetal bovine serum (FBS, BI Israel) was used for cells culturing at 37°C, 95% humidity and 5% CO_2_. Lipofectamine 3000 (ThermoFisher Scientific) was used for cell transfection. Constructs purchased from GenePharma (Shanghai) were utilized for the miRNA agonist and antagonist transfection steps, at a concentration of 200 mmol/L concentration. For transfection with siRNA NC and siRNA oligos (RiboBio), the same approach was adopted at a concentration of 50 nmol/L.

### 2.2 CCK8 assay

We use 96‐well plates (Corning) to culture cells (5 × 10^3^) for 24, 48 or 72 hours, respectively, followed by treating the cells with serum‐free medium and CCK‐8 reagent (#C0038, Beyotime) for 2 hours. The assessment of cell proliferation was carried out via measuring absorbance at a wavelength of 450 nm.

### 2.3 Transwell migration assay

Transwell inserts having 24 wells (#140629, ThermoFisher) and bearing filters with 8 μm pore size were used to carry out this assay. The upper chamber was plated with a low concentration serum medium (5%, FBS) with HUVECs (1 × 10^4^ cells per well). A total of 500 μL complete medium with 10% FBS and different treatments were added to it and were plated into the lower chamber. Cotton swabs were used to scrape off cells adhering to the filter membrane’s upper surface after 12 hours of incubation. The migrated cells that had arrived on the filter’s bottom side were then stained using crystal violet 0.5% and were counted using an optical microscope.

### 2.4 Tube formation assay

Each well was transferred into 50 μL cold Matrigel (#354234, Corning) and allowed to incubate for 50 minutes at 37°C. Matrigel‐coated 96‐well plates were taken and 2 × 10^4^ HUVECs were added into each well. Afterwards, a random assignment of cells was made to different groups based on different treatments. The cells were then cultured for 8 hours and three fields of view were obtained randomly using an inverted microscope. ImageJ software (National Institutes of Health (NIH)) was used to quantify total branch points and tube length.

### 2.5 Luciferase reporter assay

Using version 7.0 of TargetScan (http://www.targetscan.org/vert_70/), the position 1548‐1555 of 3′UTR of *PIK3R3* mRNA was determined as bearing the presumed target site of miR‐24‐3p. Its ligation into the pGL3‐basic vector (Promega Corporation) was carried out following PCR‐based amplification from HUVECs cDNA. Using Quick ChangeSite‐Directed Mutagenesis kits (Agilent Technologies, Inc), mutations at two miR‐24‐3p potential target sites were introduced for creating a pGL3‐*PIK3R3*‐3 UTR‐mutant (Mut). Next, using Lipofectamine^®^ 3000 (Thermo Fisher Scientific, Inc), co‐infection of pGL3‐*PIK3R3*‐3’UTR‐wild‐type (W; 200 ng) or pGL3‐*PIK3R3*‐3′UTR‐Mut (200 ng) was done into HUVECs, alongside the *Renilla* plasmid. miR‐NC mimic (10 nmol/L) or miR‐24‐3p mimic (10 nmol/L) was then transfected at 37°C for 48 hours. The transfection kits for the mimics were furnished by Shanghai GenePharma Co., Ltd. The relative luciferase activity of individual wells was estimated using the dual‐luciferase reporter assay system obtained from Promega Corporation. Renilla luciferase activity was normalized to Firefly luciferase expression.

### 2.6 qRT‐PCR analysis

Isolation of total RNA from samples of cells and tissues was carried out using TRIzol^®^ reagent obtained from Thermo Fisher Scientific, Inc, followed by the reverse transcription of purified RNA into cDNA using EntiLink™ 1st Strand cDNA Synthesis Kit obtained from ELK Biotechnology, EQ003. RT reaction was conducted at 42°C for 15 minutes and later at 98°C for 5 minutes while keeping the reaction volume at 20 µL. Thermocycling conditions for qPCR were as follows: initial denaturation, 30 seconds at 95°C; 40 cycles at 95°C for 5 seconds and 60°C for 30 seconds, while keeping the reaction volume as 25 µL. The internal control was GAPDH, and relative miRNA expression levels were normalized to it. $2^{‐\Delta \Delta C_{t}}$ method was used to make the calculations. The primer sequences are listed below:

miR‐24‐3p, forward, UGGCUCAGUUCAGCAGGAACAG, reverse, GUUCCUGCUGAACUGAGCCAUU; U6, forward, CTCGCTTCGGCAGCACA, reverse, AACGCTTCACGAATTTGCGT; Bcl‐2, forward, GATAACGGAGGCTGGGATGC, reverse, TCACTTGTGGCCCAGATAGG; Bax, forward, CCCTTTTGCTTCAGGGTTTC, reverse, GAGACACTCGCTCAGCTTCTTG; cyclin D1, forward, TTGCCCTCTGTGCCACAGAT, reverse, TCAGGTTCAGGCCTTGCACT; cyclin D3, forward, CTGGCCATGAACTACCTGGA, reverse, CCAGCAAATCATGTGCAATC; PIK3R3, forward, ATGTACAATACGGTGTGGAGTATG, reverse, GCTGGAGGATCCATTTCAAT; GAPDH, forward, CCGTTGAATTTGCCGTGA, reverse, TGATGACCCTTTTGGCTCCC.

### 2.7 Western blotting

Total proteins were extracted from HUVEC employing radio immunoprecipitation assay lysis buffer (cat. no. AS1004) obtained from Aspen Pharmacare Holdings Ltd. Cell lysates (1 × 10^4^) were treated with 10% SDS‐PAGE and the bicinchoninic acid method was employed to estimate protein concentration. Proteins (50 µg) were then placed onto a 10% SDS‐PVDF membrane. Bovine serum albumin (5%, Abcam) was used to block the PVDF membrane at room temperature for 2 hours. Proteins were then visualized using a chemiluminescent detection system (Canon, Inc; cat. no. LiDE110). Following antibodies were used in the Western blotting analysis: anti‐TSG 101 (1:1000; cat. no. Ab125011; Abcam), anti‐CD9 (1:1000; cat. no. Ab92726; Abcam), anti‐PIK3R3 (1:500; cat. no. Ab238509; Abcam), anti‐cyclin D1 (1:1000; cat. no. Ab40754; Abcam), anti‐cyclin D3 (1:1000; cat. no. Ab112034; Abcam) and anti‐GAPDH (1:10 000; cat no. ab37168; Abcam). Measurements were made in triplicate for all experiments.

### 2.8 Flow cytometry

An apoptosis detection kit, annexin V‐FITC/PI obtained from eBioscience, was employed to assess cell apoptosis. Following staining protocols as per the provided instructions, the cells were examined via flow cytometry.

### 2.9 Exosome isolation

Informed consent of the volunteers was obtained from the volunteers before the initiation of this study. Ethics Committee of Wuhan Union Hospital, Tongji Medical College, Huazhong University of Science and Technology approved the entire protocol of the experiments. Peripheral blood or whole blood of non‐diabetes foot wound patients, as well as DFU patients (n = 10, per group), was collected. Any possible effects of gender were eliminated using peripheral blood only from male patients. Thus, exosomes were collected from males aged between 45 and 60. Blood was collected in citrate phosphate dextrose containing tubes and spun for 15 minutes at 3000 *g*. Removal of any leftover platelets was ensured by collecting and spinning the serum again under identical conditions. Supernatants were spun for 30 minutes at 10 000 *g* followed by ultracentrifuging for 70 minutes at 100 000 *g*. After being washed thrice with phosphate‐buffered saline (PBS), the exosome pellets were ultracentrifuged under the same conditions and then resuspended in 15 mL PBS. Later exosome pellets were filtered with a 0.2‐µm filter (122‐0020PK ThermoFisher Scientific). Ultrafiltration of the samples (15 mL) was then carried out using an Amicon Ultra‐15 Centrifugal Filter (Millipore) at 4000 *g* to obtain a 200 µL net volume. The purified exosomes were then mixed with osmium tetroxide (4%) at 4°C for 30 minutes in a 50 µL volume, following which they were transmitted to a copper grid. Then, 1% phosphotungstic acid was used to stain them and their morphology was examined using a transmission electron microscope (TEM; EFI, TECNAI G2). DLS analyses were completed using a Malvern Nanosizer™ instrument (Malvern). Western blotting was used to examine the expression of exosome surface markers. Exosomes collected from individual volunteers (n = 10, diabetic vs normal) were examined individually. To measure miR‐24‐3p level in exosomes, exosomes from all people were collected, and using qRT‐PCR, individual analysis of the level of miR‐24‐3p was carried out. Additional cell and animal experiments were carried out by pooling the exosomes together for individual groups.

### 2.10 Exosome uptake assay

Tracking of the purified exosomes was accomplished by staining their membranes using PKH26 red dye (Sigma‐Aldrich). Washing of the labelled exosomes was made thrice using PBS, and exosomes were recollected by ultracentrifugation. HUVEC uptake of the labelled exosomes was evaluated by combining exosomes and cells, and the uptake was quantified using immunofluorescence.

### 2.11 Animal wound model and administration

Research protocols opted during this work followed the guidelines for Laboratory animal care and use and duly approved by the Wuhan Union Hospital Animal Care Committee, Tongji Medical College, Huazhong University of Science and Technology. Male C57BL/6J mice (weighing 25‐35 g and 8 weeks old) were obtained from The Center of Experimental Animal, Tongji Medical College, Huazhong University of Science and Technology. Mice were single‐caged and housed at room temperature of 18°C with a 12/12‐hour light‐dark cycle. They had free access to water and were fed with a chow diet. Animals were anaesthetized using an intraperitoneal injection (i.p.) of 10% chloral hydrate (300 mg/kg bodyweight). No signs of peritonitis, pain or discomfort were observed after anaesthesia. After shaving the mice, the upper back of individual mice was given one full‐thickness skin excision with a diameter of 10 mm followed by the sterilization of the wound area with 1% polyvinylpyrrolidone. Five groups of wounded mice were randomly defined: (a) Control group where 100 µL PBS was used for wound treatment); (b) Con‐Exos group in which 200 μg Con‐Exos in 100 µL PBS was used for wound treatment); (c) Dia‐Exos group in which 200 μg Dia‐Exos in 100 µL PBS was used for wound treatment; (d) AgomiR‐24‐3p group in which 2 OD AgomiR‐24‐3p in 100 µL diethylpyrocarbonate (DEPC) water was used for wound treatment); and (e) AntagomiR‐24‐3p group in which 2 OD AntagomiR‐24‐3p in 100 µL DEPC water was used for wound treatment. On 0, 3, 5, 7, 9 and 11 post‐wounding days (n = 6), subcutaneous injections were given to the mice at four injection sites around the wound (25 µL per site). A transparent dressing (Tegader TM Film) was used to cover and protect the wound.

Photographs of the wounds were taken at 0, 3, 5, 7, 10, and 14 post‐injury days. On the same day, measurement of the wound was taken using a calliper ruler. The percentage of wound closure was then calculated using ImageJ software by the formula below.

Cn=(A0‐An)/A0×100%

“Cn” is the percentage of wound area reduction at day 3, day 5, day 7, day 10 and day 14 after injury, “A0” is the original wound area, and “An” represents the wound area at day 3, day 5, day 7, day 10 and day 14 after injury.

### 2.12 Small animal Doppler

After 10 days of the operation, the laser speckle contrast imaging (LSCI) system was applied for examining the state of local blood flow or more accurately blood perfusion. A PSI‐ZR PeriCam system which is a product of PERIMED Ltd, was employed for obtaining images of wounds. Blood perfusion measurements were carried out using an invisible near‐infrared laser with 785 nm wavelength, and perfusion units were determined accordingly. Wounds were photographed while maintaining a constant distance from the surface of the wound and keeping the same scan area dimensions the same each time. PIMSoft (Moor Instruments Ltd) was employed to calculate the mean perfusion units (MPU) ratio, obtained by correlating the MPU at wound area (ROI‐1) to the MPU in the region surrounding the wound (ROI‐2), using flux images of individual wound sites.

### 2.13 Haematoxylin and eosin staining

Thick tissue samples (7 μm) embedded with paraffin were prepared and treated with an Haematoxylin and eosin (H&E) stain. Measurement and imaging of sections were carried out using a DP73 Olympus CCD Imaging System and an Olympus BX51 microscope (Olympus Corporation).

### 2.14 Statistical analysis

All experimental data were presented as means ± SD. A total of two groups were compared using Student’s *t* tests whereas when needed ANOVA with post hoc Tukey was employed to analyse more than two groups when needed. GraphPad prism 8.0 (GraphPad Software, La Jolla, CA) was used for the analysis of the data. *P* < .05 was considered significant.

## 3 RESULTS

### 3.1 Con‐Exos and Dia‐Exos, exploring the characteristics

**Figure 1:**
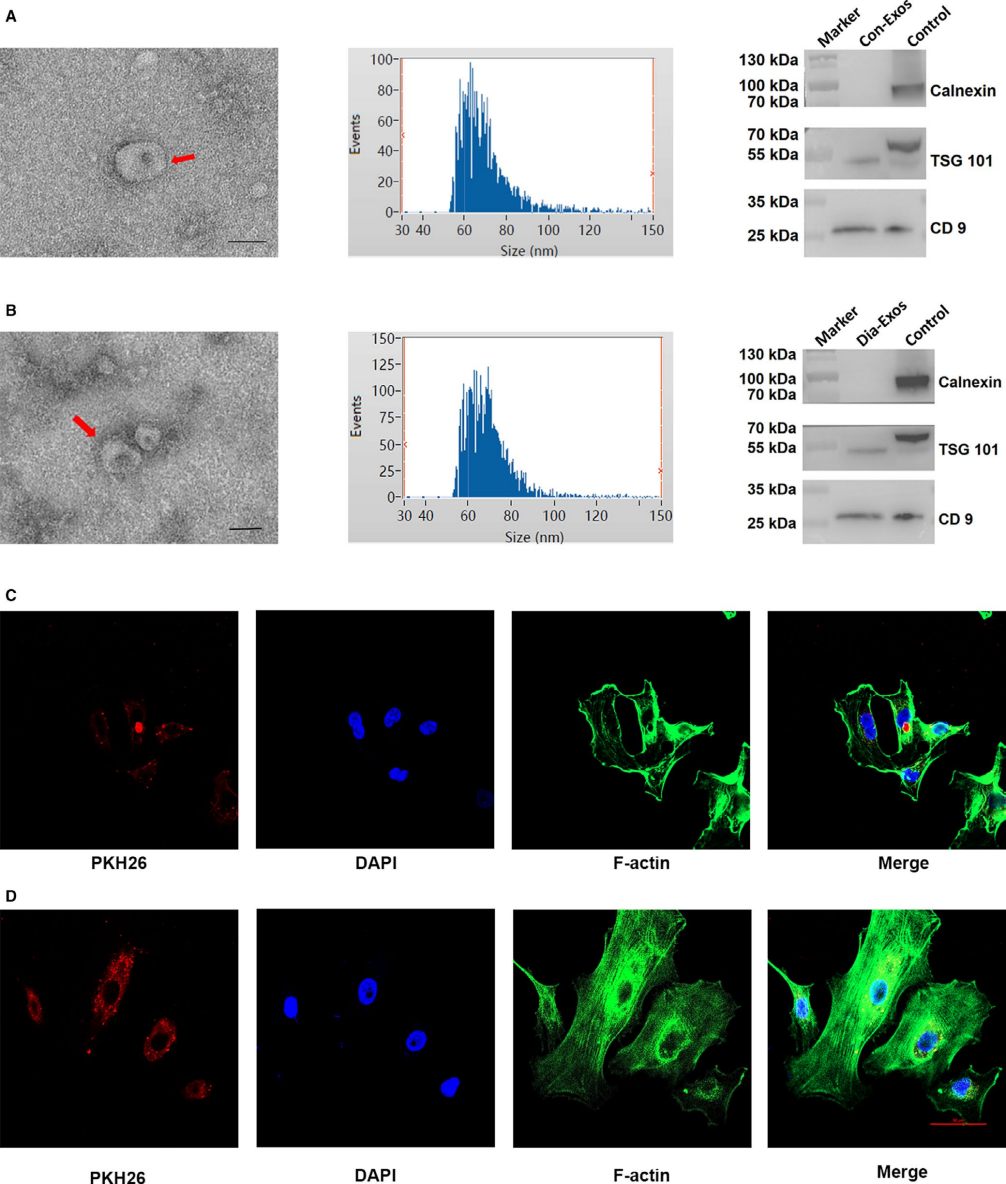
Characteristics of Con‐Exos and Dia‐Exos. A, The DLS result, TEM images and WB analysis of Con‐Exos; (B) the DLS result, TEM images and WB analysis of Dia‐Exos, scale bar, 100 nm; (C) PKH26‐labelled Con‐Exos were absorbed by HUVECs as indicated by a red fluorescent signal; (D) PKH26‐labelled Dia‐Exos were uptaken by HUVECs as indicated by a red fluorescent signal

The characteristic features of Con‐Exos and Dia‐Exos were identified using TEM, DLS and Western blotting. Data acquired from TEM and DLS results suggested that Con‐Exos were cup‐ or sphere‐shaped with a diameter ranging from 30 to 150 nm, which is similar to the data from the previous research report.^16^ The Western blotting analysis confirmed the surface markers of exosomes including CD9 and TSG 101 in Con‐Exos (Figure 1A). Consistently, similar results were observed for Dia‐Exos by DLS, TEM and Western blotting analysis (Figure 1B). Subsequently, we performed an exosome uptake assay by HUVECs in vitro while using PKH26 to label either Con‐Exos or Dia‐Exos. As seen in Figure 1C,D, Con‐Exos and Dia‐Exos were both taken up by HUVECs when they were added into the HUVECs culture medium.

### 3.2 Suppression of cutaneous wound repair in vivo by Dia‐Exos

**Figure 2:**
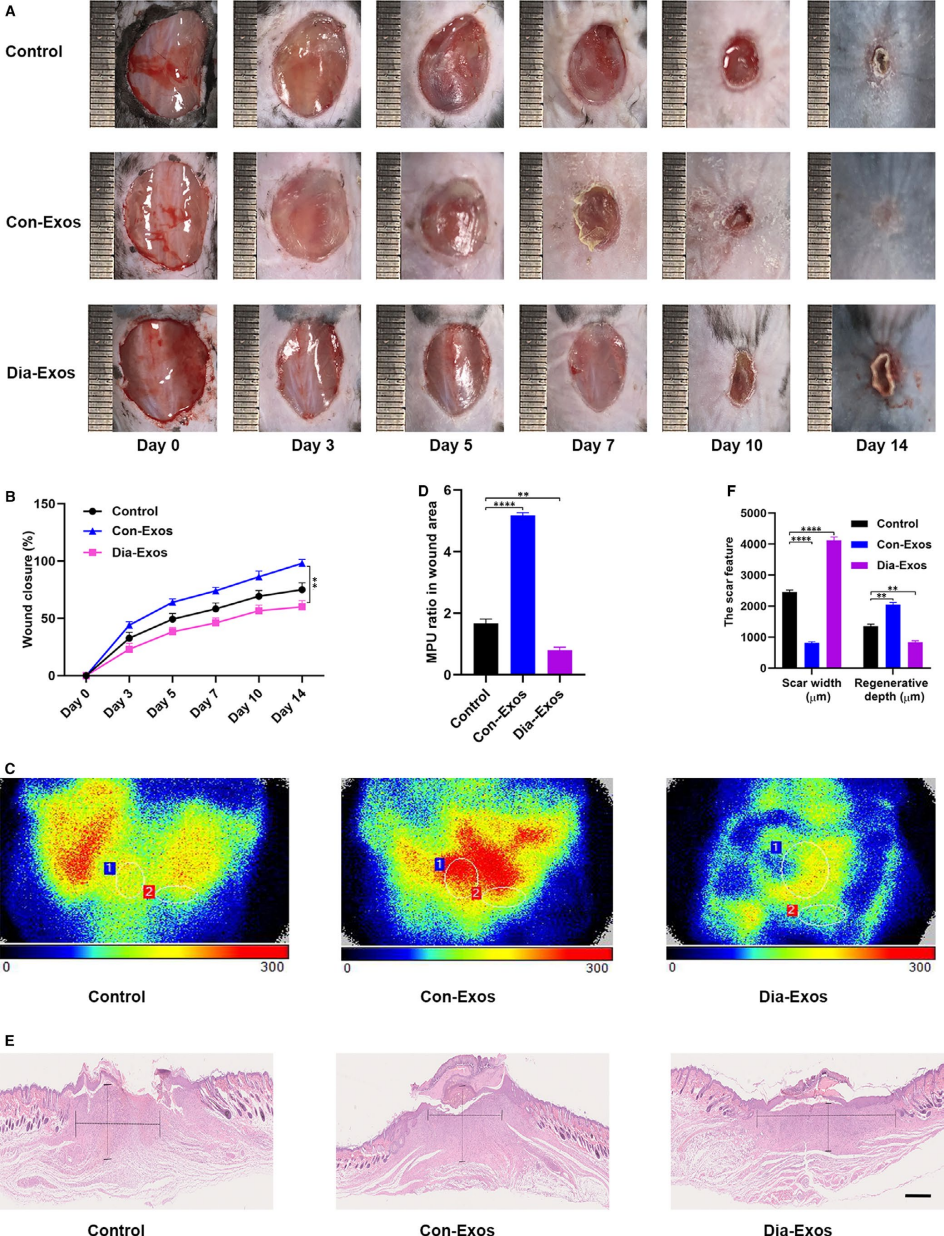
Dia‐Exos hinders wound healing in vivo. A, General view of wounds closure with different treatments at day 0, 3, 5, 7, 10 and 14 after wounding; (B) The rate of wound closure among the three groups, n = 6, per group; (C,D) The MPU ratio at the area of the wounds among the three groups was assessed by Doppler of small animals, MPU ratio is the comparative result of MPU at the wound area (ROI‐1) and the area around the wound (ROI‐2). n = 6, per group; (E,F) H&E staining of wound sections treated with PBS, Con‐Exos and Dia‐Exos at 14 d after the operation. Scale bar: 500 μm. Data are the means ± SD three independent experiments. *P < .05, **P < .01, ***P < .001

The effects of Con‐Exos and Dia‐Exos on wound repair in murine models were explored in detail. Cutaneous wounds with full thickness were produced on mice’s backs. Local injections were then administrated. Mice were randomly categorized into three separate groups based upon the different treatments they received. Although the control group was given PBS, Con‐Exos and Dia‐Exos groups were given Con‐Exos and Dia‐Exos, respectively. The volume of treatment given was kept similar for all groups. An assessment of the general view of wounds was first made and the results indicated a significantly slower wound closure rate in the Dia‐Exos group as evident in Figure 2A,B. Next, the perfusion of blood in the wound area was compared using small animal Doppler. The results suggested that in the Dia‐Exos group, relative to other groups, a significant decrease in the MPU ratio was observed on the location of the wound, following 10 days of wound introduction (Figure 2C,D). In addition, the wound site was stained with H&E stain to assess scar formation and the degree of re‐epithelialization. Only much shorter newly formed epidermis and dermis were observed in the wound site in the Dia‐Exos group, in comparison with the PBS‐treated and Con‐Exos‐treated groups on day 14 after wounding (Figure 2E). Quantitative analysis revealed that the wounds treated with Dia‐Exos had a significantly lower re‐epithelialization rate and a greater extent of scar formation compared with other groups (Figure 2F).

### 3.3 Dia‐Exos impaired HUVECs angiogenesis and survival

**Figure 3:**
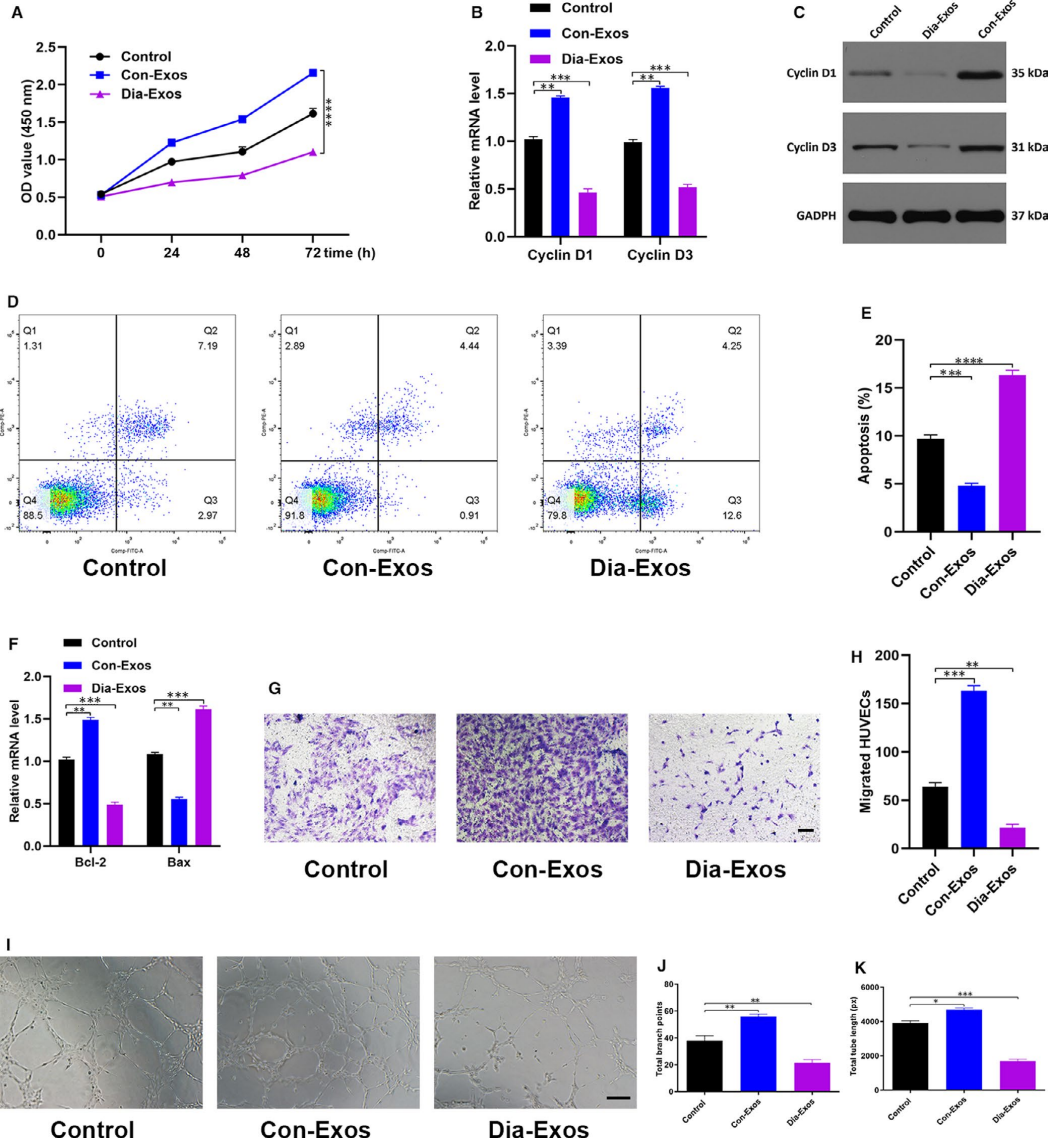
Dia‐Exos inhibit HUVECs’ angiogenesis and survival in vitro. A, Effect of Dia‐Exos on HUVECs’ proliferation measured by CCK‐8 assay; (B,C) the effect of Dia‐Exos on the proliferation‐related mRNAs Cyclin D1 and Cyclin D3 levels assessed by qRT‐PCR and Western blotting; (D,E) flow cytometry was further used to quantify cell apoptosis, showing a greater percentage of HUVECs was in G1 phase and apoptotic following Dia‐Exos treatment; (F) the effect of Dia‐Exos on the apoptosis‐related mRNAs Bcl‐2 and Bax levels assessed by qRT‐PCR analysis; (G,H) the effect of Dia‐Exos on HUVEC migration was assessed by transwell migration assay, scale bar, 100 µm; (I‐K) effects of Dia‐Exos on the angiogenesis ability of HUVECs was measured by tube formation assay, scale bar, 200 µm. Data are the means ± SD three independent experiments. *P < .05, **P < .01, ***P < .001

Dia‐Exos influence on HUVECs functionality was evaluated. Cell counting kit‐8 (CCK8) proliferation assay showed significantly decreased HUVEC proliferation by Dia‐Exos relative to the other treatments (Figure 3A). *Cyclin D1* and *Cyclin D3*, which are categorized as the primary proliferation‐related proteins, were found to reduce in number in HUVECs treated with Dia‐Exos which is consistent with the CCK8 assay result (Figure 3B,C). Further quantification of cell apoptosis was made using flow cytometry which revealed that following Dia‐Exos treatment, a major percentage of HUVECs was found to be apoptotic in the G1 phase (Figure 3D,E). Reduced expression of *Bcl‐2* and a simultaneous elevated expression of *Bax* was also evident after treatment with Dia‐Exos (Figure 3F). Transwell migration assay was further carried out and the result indicated a weakened migration ability of HUVECs following treatment with Dia‐Exos compared with the other groups (Figure 3G,H). Moreover, tube formation assay examined the Dia‐Exos’ influence on the angiogenesis of HUVEC which showed a significant decrease in the formation of tube following Dia‐Exos treatment (Figure 3I,K).

### 3.4 MiR‐24‐3p is rich in Dia‐Exos and regulates HUVECs functionality

**Figure 4:**
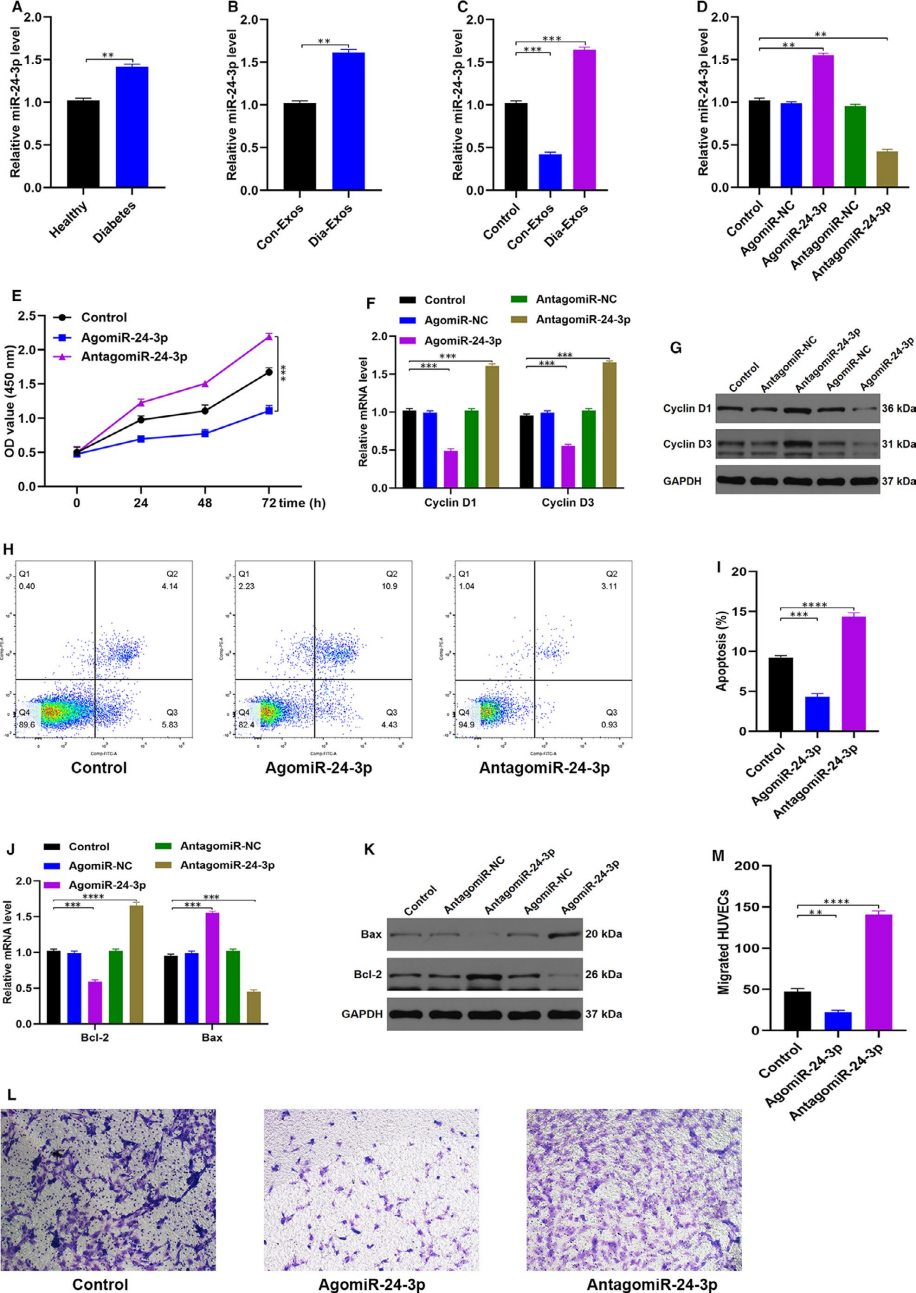
MiR‐24‐3p is enriched in Dia‐Exos and modulate HUVEC function. A, The overexpression of miR‐24‐3p was found in the serum samples of diabetes group, n = 10, per group; (B) the overexpression of miR‐24‐3p was found in the exosomes of diabetes group, n = 10, per group; (C) the overexpression of miR‐24‐3p was found in the murine newly formed tissue 14 d after injury of Dia‐Exos‐treated group, n = 6, per group; (D) qRT‐PCR indicated that usage of AntagomiR‐24‐3p was able to induce the overexpression of miR‐24‐3p; (E) effect of AntagomiR‐24‐3p on HUVECs’ proliferation measured by CCK‐8 assay; (F,G) the effect of AntagomiR‐24‐3p on the proliferation‐related mRNAs cyclin D1 and cyclin D3 levels assessed by qRT‐PCR and Western blotting; (H,I) Flow cytometry was further used to quantify cell apoptosis, showing a smaller percentage of HUVECs were in G1 phase following AntagomiR‐24‐3p treatment; (J,K) the effect of AntagomiR‐24‐3p on the apoptosis‐related mRNAs Bcl‐2 and Bax levels assessed by qRT‐PCR analysis and Western blotting; (L,M) the effect of AntagomiR‐24‐3p on HUVEC migration was assessed by transwell migration assay, scale bar, 100 µm. Data are the means ± SD three independent experiments. *P < .05, **P < .01, ***P < .001

Relatively new research shows that salivary exosomal miR‐24‐3p is overexpressed in elderly people.^17^ The incidence of diabetes, especially for type 2 diabetes, is also rather high in this age group. Thus, we measured the miR‐24‐3p level both in the Con‐Exos and in the Dia‐Exos. As shown in Figure 4A and Figure 4B, qRT‐PCR (quantitative reverse transcription‐polymerase chain reaction) analysis confirmed that as compared to non‐diabetics, the serum and circulating exosomes of diabetics show a raised miR‐24‐3p level. Similarly, skin tissues with Dia‐Exos treatment showed overexpression of miR‐24‐3p (Figure 4C). Our work thus proceeded to look at the impact of miR‐24‐3p on the proliferation, apoptosis and migration of HUVECs. The HUVECs were first transfected with miR‐24‐3p agonist or antagonist. The qRT‐PCR analysis revealed that the miR‐24‐3p level was notably high in the agonist group whereas there was a significantly decreased amount of miR‐24‐3p in the antagonist group (Figure 4D). Furthermore, the results from CCK8 cell proliferation assays pointed out that miR‐24‐3p agonist treatment notably impaired HUVEC proliferation (Figure 4E). *Cyclin D1* and *Cyclin D3*, the proliferation‐related proteins, were found in a decreased number in HUVECs exposed to the miR‐24‐3p agonist. This observation is consistent with the CCK8 assay result (Figure 4F,G). Moreover, the quantification of cell apoptosis was made using flow cytometry which revealed a large proportion of HUVECs was found apoptotic in the G1 phase of the cell cycle following miR‐24‐3p agonist treatment (Figure 4H,I). The diminished expression of *Bcl‐2* and the elevated expression of *Bax* after treatment with miR‐24‐3p agonists were also consistent with this observation (Figure 4J,K). In addition, transwell migration assay results showed that HUVECs had only a weakened migration ability after being treated with miR‐24‐3p agonist (Figure 4L,M).

### 3.5 Knocking down miR‐24‐3p can partially reverse the impaired effect of Dia‐Exos

**Figure 5:**
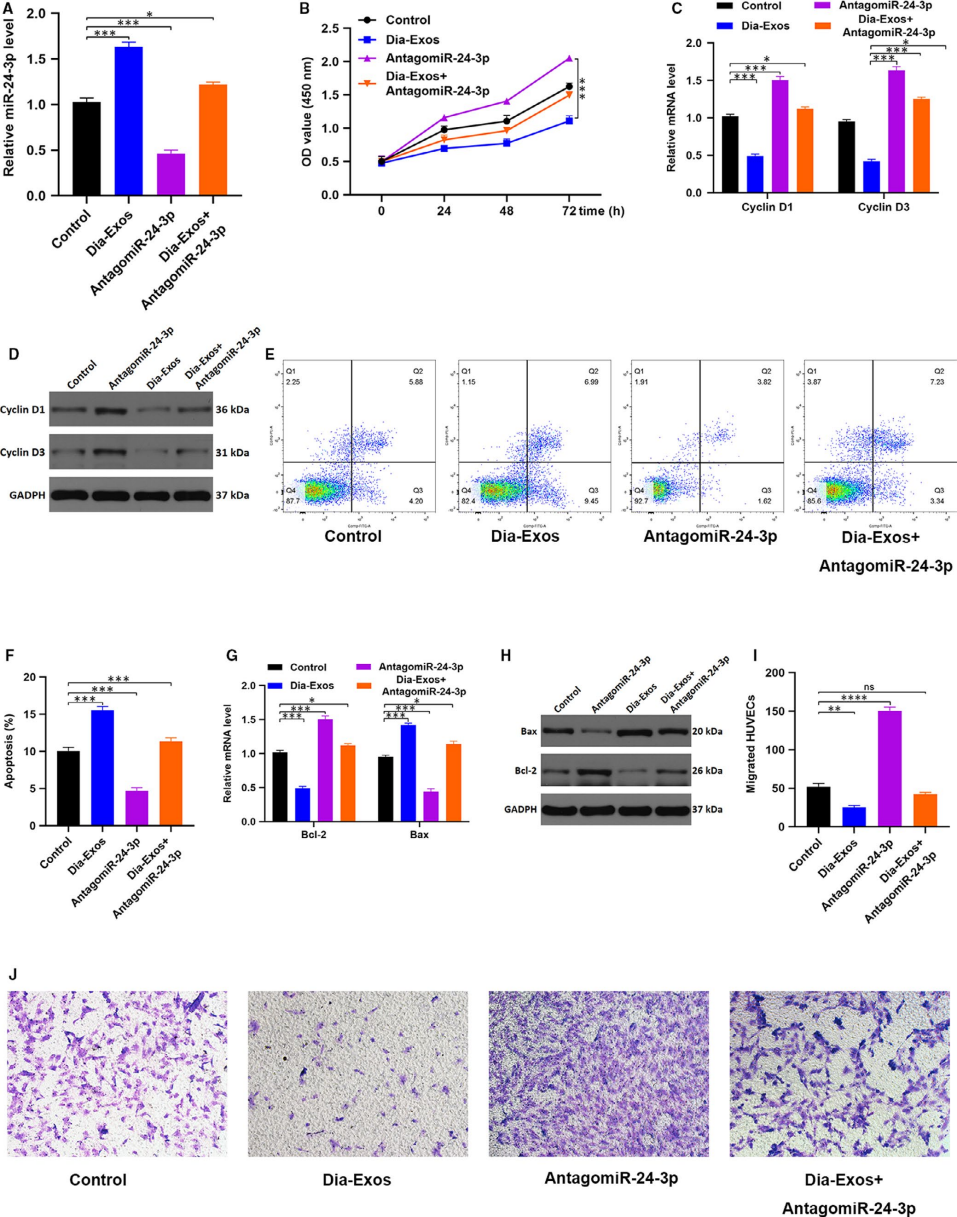
Reduction of miR‐24‐3p can partially reverse the Dia‐Exos impaired effect of HUVECs. A, miR‐24‐3p level among different groups measured by qRT‐PCR analysis; (B) CCK‐8 assay result of the differently treated groups; (C,D) The proliferation‐related mRNAs cyclin D1 and cyclin D3 levels in different treated HUVECs were assessed by qRT‐PCR and Western blotting; (E,F) flow cytometry was further used to quantify cell apoptosis in different treated groups; (G,H) the apoptosis‐related mRNAs Bcl‐2 and Bax levels were assessed by qRT‐PCR analysis and Western blotting; (I,J) HUVEC migration ability was assessed by transwell migration assay, scale bar, 100 µm. Data are the means ± SD three independent experiments. *P < .05, **P < .01, ***P < .001

The miR‐24‐3p expression in Dia‐Exos was knocked down using an antagonist of miR‐24‐3p. Depending upon the type of treatment, three categories were defined, including the PBS group (Control), Dia‐Exos group and Dia‐Exos + AntagomiR‐24‐3p group. The qRT‐PCR analysis indicated that compared with the single Dia‐Exos treated group, a significant decreased miR‐24‐3p level was observed for the Dia‐Exos + AntagomiR‐24‐3p group (Figure 5A). Later, CCK8 cell proliferation assays were carried out to evaluate HUVECs proliferation among the different groups. The result showed an increased proliferation of HUVECs following Dia‐Exos + AntagomiR‐24‐3p treatment in comparison with the single Dia‐Exos treated group (Figure 5B). Similarly, *Cyclin D1* and *Cyclin D3,* the proliferation‐related proteins, were seen to be partially recovered after adding miR‐24‐3p antagonist treatment (Figure 5C,D). Furthermore, quantification of cellular apoptosis via flow cytometry revealed only a smaller percentage of HUVECs in the G1 phase of the cell cycle and apoptotic after Dia‐Exos + AntagomiR‐24‐3p treatment than the single Dia‐Exos treatment (Figure 5E,F). After miR‐24‐3p antagonist treatment, *Bcl‐2* expression was found to be increased and *Bax* expression was found to be decreased (Figure 5G,H). In addition, a transwell migration assay was performed and the result showed that migration ability of Dia‐Exos‐treated ‐HUVECs was partially reversed after adding miR‐24‐3p agonist (Figure 5I,J).

### 3.6 MiR‐24‐3p regulates HUVEC functionality via directly targeting *PIK3R3*

**Figure 6:**
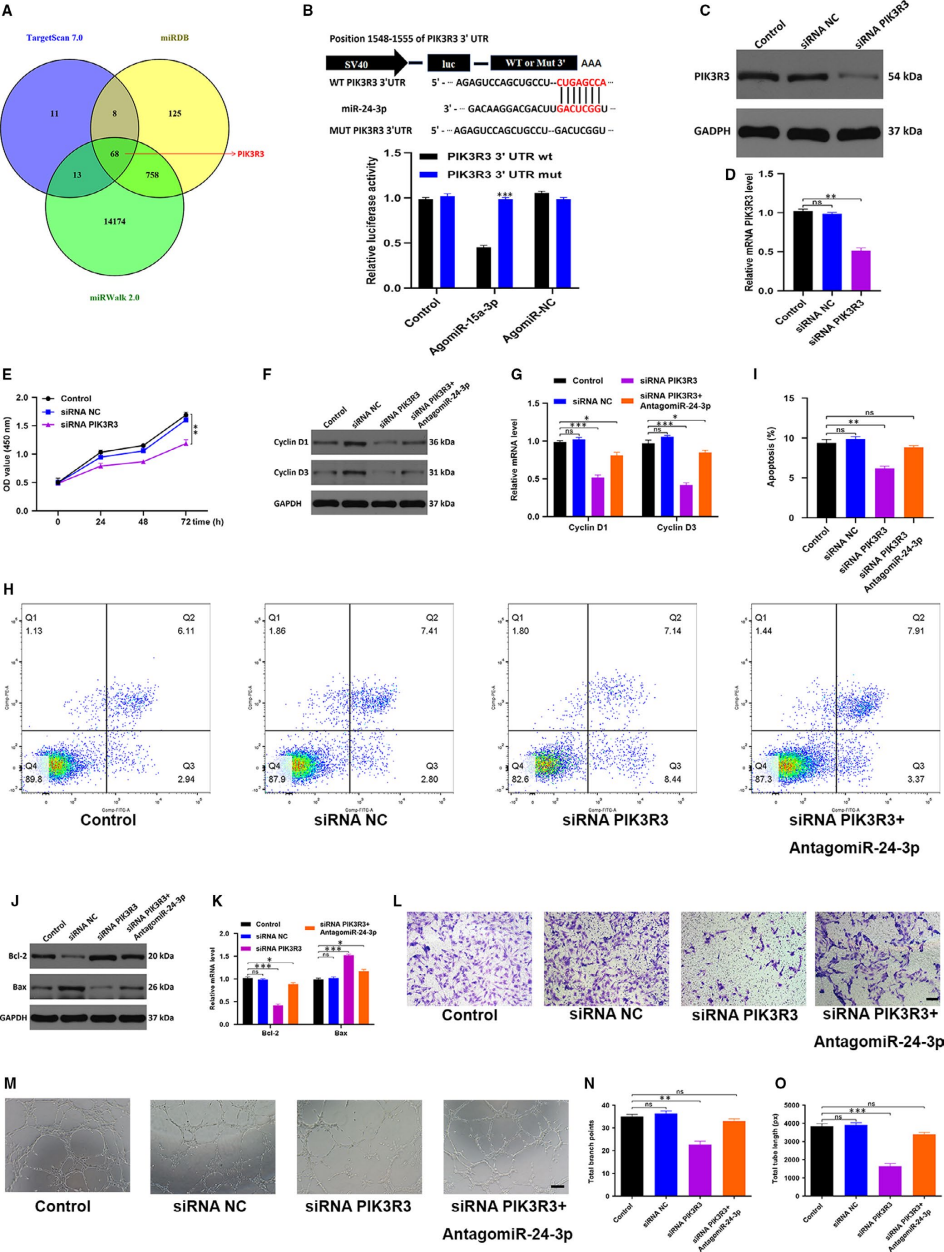
MiR‐24‐3p directly targets PIK3R3. A, Putative miR‐24‐3p targets were identified via using miRbase, Targetscan and miRwalk online predicting tools, and 68 genes were identified as potential miR‐24‐3p target genes; (B) the association between miR‐24‐3p and PIK3R3 was demonstrated by luciferase reporter assay; (C,D) Western blotting and qRT‐PCR analyses were used to detect the efficacy of siRNA NOX5; (E) CCK8 assay was applied to assess the cell proliferation with different treatments; (F,G) The qRT‐PCR and Western blotting results of the proliferation‐related mRNAs cyclin D1 and cyclin D3; (I,J) flow cytometry was further used to quantify cell apoptosis in treated cells; (J,K) the apoptosis‐related mRNAs Bcl‐2 and Bax levels were measured by qRT‐PCR and Western blotting analysis; (L) the effect of siRNA PIK3R3 on HUVEC migration was assessed by transwell migration assay, scale bar, 100 µm; (M‐O) effects of siRNA PIK3R3 on the angiogenesis ability of HUVECs was measured by tube formation assay, scale bar, 200 µm. Data are the means ± SD three independent experiments. *P < .05, **P < .01, ***P < .001

We subsequently investigated the mechanisms regulating the inhibition of HUVEC functionality by miR‐24‐3p. Our research was directed towards finding putative miR‐24‐3p targets using Targetscan, miRbase and miRanda, which eventually led us to one target gene worth validation namely *PIK3R3* (Figure 6A). We attempted to verify the correlation between *PIK3R3* and miR‐24‐3p using Luciferase assay and the outcome indicated that miR‐24‐3p links specifically to *PIK3R3* mRNA at the predicted target region (Figure 6B). We then tested the biological function of *PIK3R3* on HUVECs by transfecting HUVECs with siRNA specific for *PIK3R3*. We found that the siRNA specific for *PIK3R3* caused a significant decrease in *PIK3R3* expression in treated cells (Figure 6C,D). CCK8 cell proliferation assay was also carried out and the result indicated that siRNA *PIK3R3* could inhibit HUVEC proliferation (Figure 6E). Similarly in HUVECs treated with siRNA *PIK3R3, Cyclin D1* and *Cyclin D3* proteins, which take part in mediating cell cycle, were depleted, which is in good agreement with the findings from CCK8 (Figure 6F,G). Flow cytometry measured the extent of cell apoptosis and found the majority of HUVECs in the G1 phase of cell cycle and apoptotic following siRNA *PIK3R3* treatment (Figure 6H,I). Diminished *Bcl‐2* expression of and an elevated *Bax* expression was also subsequently found following siRNA *PIK3R3* treatment (Figure 6J,K). Transwell migration assay was further carried out, and the result indicated a weakened migration ability of HUVECs after treated with siRNA *PIK3R3* compared with the other groups (Figure 6L). Additionally, the effect of siRNA *PIK3R3* on the angiogenesis of HUVEC was evaluated using tube formation assay, which revealed that the extent of formation of the tube was significantly decreased after siRNA *PIK3R3* treatment (Figure 6M‐O).

### 3.7 miR‐24‐3p delayed wound healing in vivo

**Figure 7:**
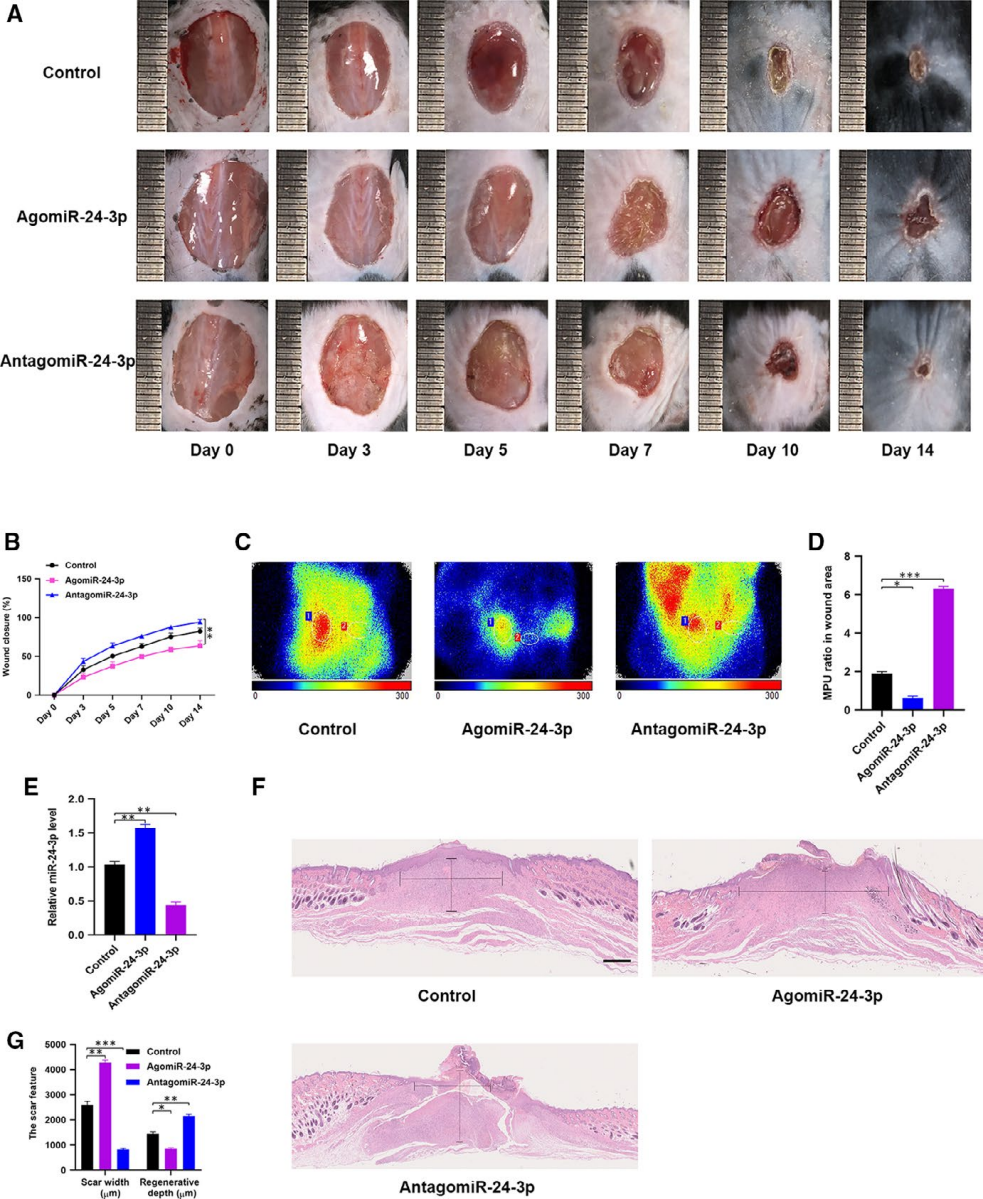
miR‐24‐3p delays wound healing in vivo. A, General view of wounds closure with different treatments at day 0, 3, 5, 7, 10 and 14 after wounding; (B) the rate of wound closure among the three groups, n = 6, per group; (C,D) the MPU ratio at the area of the wounds among the three groups was assessed by Doppler of small animals, MPU ratio is the comparative result of MPU at the wound area (ROI‐1) and the area around the wound (ROI‐2). n = 6, per group; (E) the miR‐24‐3p level was measured among the different groups by qRT‐PCR analysis; (F,G) H&E staining of wound sections treated with PBS, AgomiR‐24‐3p and AntagomiR‐24‐3p at 14 d after the operation. Scale bar: 500 μm. Data are the means ± SD three independent experiments. *P < .05, **P < .01, ***P < .001

The effects of miR‐24‐3p on wound repair in murine models were explored in detail. Cutaneous wounds with full thickness were produced on mice’s backs. Local injections were then administrated. Mice were randomly categorized into three separate groups based upon the different treatments they received. The control group was given PBS, whereas AgomiR‐24‐3p and AntagomiR‐24‐3p groups were given AgomiR‐24‐3p and AntagomiR‐24‐3p, respectively. The volume of treatment given was kept similar for all groups. An assessment of the general view of wounds was first made and the results indicated a significantly slower wound closure rate in the AgomiR‐24‐3p group as evident in Figure 7A,B. Next, the perfusion of blood in the wound area was compared using small animal Doppler. The results suggested that in the AgomiR‐24‐3p group, relative to other groups, a significant decrease in the MPU ratio was observed on the location of the wound, following 10 days of wound introduction (Figure 7C,D). The level of the skin tissue was measured after euthanasia of the mice, and the qRT‐PCR result indicated an elevated level of miR‐24‐3p in the AgomiR‐24‐3p group (Figure 7E). In addition, the wound site was stained with H&E stain to assess scar formation and the degree of re‐epithelialization and only a much shorter newly formed epidermis and dermis were observed in the wound site in the AgomiR‐24‐3p group, in comparison with the PBS‐treated and AntagomiR‐24‐3p‐treated groups at day 14 after wounding (Figure 7F). Quantitative analysis revealed that the wounds treated with AgomiR‐24‐3ps had a significantly lower re‐epithelialization rate and a greater extent of scar formation compared with other groups (Figure 7G).

## 4 DISCUSSION

In the few past decades, a variety of advanced therapies for wound healing have attracted accumulative attention among scientists and clinicians.^18^ For example, a recent study reported that a significant number of proteins with a potential role in wound healing are found in the saliva and salivary‐VEGF plays a noteworthy role in mucosal wound healing. This finding provides a sound basis for developing novel therapeutics with an enhanced potential of wound repair in the oral mucosa as well as other regions in the alimentary canal.^19^ It has been shown that the quality and levels of expression of RNA and DNA obtained from serum were comparable to those in other body fluids or cellular samples.^20^ Furthermore, recent research has revealed a significant difference in circulating exosomal miR‐20b‐5p between diabetics and non‐diabetic individuals.^14^ Therefore, we aimed to explore the significantly expressed miRNAs between Con‐Exos and Dia‐Exos. In the present study, we found Con‐Exos and Dia‐Exos had a different effect on wound healing, and miR‐24‐3p was found to be rich in Dia‐Exos, and inhibition of this miRNA could partially reverse the negative effect of Dia‐Exos. Thus, our results elucidated the regulatory role of Dia‐Exos in wound healing and provided evidence that the knocking down of circulating miR‐24‐3p might have therapeutic applications in the treatment of DFU.

Exosome, an extracellular vesicle with a diameter of 30‐100 nm, has a strong biological function. The vesicle contains non‐coding RNA, cholesterol, protein and other bioactive substances, which have extensively proved to regulate biological processes in vivo.^21^, ^22^ Due to their protective functions for their contents such as proteins, non‐coding RNAs and nucleic acids, their ability to deliver them to target cells, and their vital role in paracrine and endocrine type messaging between organs and cells, exosomes have surfaced as perfect drug carriers for many diseases.^23^ Recent research has identified salivary exosomal miR‐24‐3p as a potential ageing biomarker,^17^ and it is evident that the incidence of diabetes, especially for type 2 diabetes, is rather high in the elderly people. The level of miR‐24‐3p both in Con‐Exos and in Dia‐Exos thus needed to be examined. Accordingly, the outcome from qRT‐PCR communicates that miR‐24‐3p is significantly increased in Dia‐Exos, and in terms of wound repair, this miRNA has been demonstrated to have a negative regulation in our in vitro as well as in vivo experiments. Interestingly, knocking down the miR‐24‐3p expression in Dia‐Exos caused a significant reversal in the adverse effect of Dia‐Exos on wound healing. Thus, we conclude that the elevated circulating exosomal miR‐24‐3p is the potential mechanism for the regulation of Dia‐Exos and is a potential target for therapeutic purposes of circulating exosome for DFU treatment. It is reported that various tissues, such as adipose tissue, muscle and bone marrow, can release exosomes into the circulation. Thus, we propose all these tissues may be the potential origins of the exosomal miR‐24‐3p in diabetes patients.^24^, ^25^, ^26^

The *PI3K/AKT* pathway plays an important role in cell proliferation and differentiation.^27^ *PIK3R3*, as a regulatory subunit of *PI3K*, presumptively plays a critical regulatory role in the biological process.^28^ However, whether or not *PIK3R3* could accelerate cutaneous wound healing is still unclear. Thus, we probed into the effect of abnormal *PIK3R3* level on the functionality of HUVECs. According to our data, overexpression of miR‐24‐3p was causing a highly limited mRNA expression of *PIK3R3,* and the reduction of *PIK3R3* inhibited HUVEC proliferation, migration, angiogenesis, as well as inducing HUVEC apoptosis. Furthermore, online predicting tools also identified that the presumed gene target of miR‐24‐3p was *PIK3R3* which was further validated by luciferase assays.

Taken together, the current work suggested that circulating exosomal miR‐24‐3p participated in the angiogenesis, survival and migration of HUVECs. It also demonstrated that *PIK3R3* was partially responsible for the miR‐24‐3p‐induced inhibition of wound repair. The key findings of this work set up a sound basis for exploring other potential applications of circulating exosomes as a novel treatment strategy for DFU.

## CONFLICT OF INTEREST

All authors declare no conflict of interest.

## AUTHOR CONTRIBUTION

**Yan Xu:** Data curation (equal); Formal analysis (equal); Writing‐original draft (equal). **Liu Ouyang:** Data curation (equal); Formal analysis (equal); Writing‐original draft (equal). **Lei He:** Investigation (equal); Methodology (equal). **Yanzhen Qu:** Formal analysis (equal); Software (equal). **Yu Han:** Validation (equal); Visualization (equal). **Deyu Duan:** Conceptualization (lead); Funding acquisition (lead); Project administration (lead).

## ETHICAL APPROVAL

Ethics Committee of Union Hospital, Tongji Medical College, Huazhong University of Science and Technology, provided the ethical approval for this study.

## Data Availability

The data that support the findings of this study are available from the corresponding author upon reasonable request.
